# The human *PINK1 *locus is regulated *in vivo *by a non-coding natural antisense RNA during modulation of mitochondrial function

**DOI:** 10.1186/1471-2164-8-74

**Published:** 2007-03-15

**Authors:** Camilla Scheele, Natasa Petrovic, Mohammad A Faghihi, Timo Lassmann, Katarina Fredriksson, Olav Rooyackers, Claes Wahlestedt, Liam Good, James A Timmons

**Affiliations:** 1 Department of Cellular and Molecular Biology, Program for Genomics and Bioinformatics, Karolinska Institutet, Stockholm, SE171 77, Sweden; 2 Department of Biochemistry, Scripps Research Institute, Florida, Jupiter, FL 33458, USA; 3 Department of Anesthesiology and Intensive Care, Karolinska University Hospital, Sweden; 4 School of Life Sciences, Heriot-Watt University, EH14 4AS Edinburgh, Scotland, UK

## Abstract

**Background:**

Mutations in the PTEN induced putative kinase 1 (*PINK1*) are implicated in early-onset Parkinson's disease. PINK1 is expressed abundantly in mitochondria rich tissues, such as skeletal muscle, where it plays a critical role determining mitochondrial structural integrity in *Drosophila*.

**Results:**

Herein we characterize a novel splice variant of *PINK1 *(svPINK1) that is homologous to the C-terminus regulatory domain of the protein kinase. Naturally occurring non-coding antisense provides sophisticated mechanisms for diversifying genomes and we describe a human specific non-coding antisense expressed at the *PINK1 *locus (naPINK1). We further demonstrate that PINK1 varies *in vivo *when human skeletal muscle mitochondrial content is enhanced, supporting the idea that PINK1 has a physiological role in mitochondrion. The observation of concordant regulation of svPINK1 and naPINK1 during in vivo mitochondrial biogenesis was confirmed using RNAi, where selective targeting of naPINK1 results in loss of the PINK1 splice variant in neuronal cell lines.

**Conclusion:**

Our data presents the first direct observation that a mammalian non-coding antisense molecule can positively influence the abundance of a *cis*-transcribed mRNA under physiological abundance conditions. While our analysis implies a possible human specific and dsRNA-mediated mechanism for stabilizing the expression of svPINK1, it also points to a broader genomic strategy for regulating a human disease locus and increases the complexity through which alterations in the regulation of the *PINK1 *locus could occur.

## Background

PTEN induced putative kinase 1 gene (*PINK1*) is a serine-threonine kinase directly linked to a recessive form of familial parkinsonism [1-4]. A mutation at the nucleotide binding site within the kinase domain renders the protein unable to protect neuroblastoma cells from apoptosis, whereas over expression of the native peptide protects SH-SY5Y cells [1,3]. Over expressed tagged PINK1 localizes to the mitochondria [2,4], raising the possibility that PINK1 phosphorylates and regulates proteins involved in oxidative phosphorylation or the mitochondrial translocator pore [5,6], processes linked to neuronal cell death [7,8]. Indeed, PINK1 inhibits mitochondrial cytochrome c release, attenuating the general apoptosis machinery [3,6], while mitochondrial localization is supported by the existence of an 8.5 kDa mitochondrial import tag [2] and *in vitro *mitochondrial translocation [2]. Most recently, Gandhi *et *al. [9] produced evidence that endogenous PINK1 protein locates to the mitochondrial membranes, while three additional articles highlighted a critical role for PINK1 in *Drosophila *flight muscle mitochondria [10-12]. A fourth study in *Drosophila *show an antioxidant rescue of neurodegeneration induced by knockdown of Pink1 [13]. It is clear, therefore, that PINK1 may play a rather central physiological role in neuronal and myocyte energy metabolism, consistent with its abundant expression in mitochondria-rich tissue [14] and consistent with the proposed mitochondrial basis for Parkinsonism.

The C-terminal end of the protein regulates PINK1 kinase activity [2]. Intriguingly, a predicted novel short splice variant of PINK1 would translate into a protein sequence which represents the C-terminus portion of the protein. Furthermore, a *cis*-transcribed non-coding natural antisense (ncNAT) originating from the *PINK1 *gene locus is predicted from the human EST databases [15] and is now, very recently, listed in a natural antisense transcript database [16]. According to the available well annotated databases, PINK1 ncNAT is not apparent in other species. The ncNAT demonstrates near complete sequence overlap with the short PINK1 splice variant and only partial overlap with PINK1, at the 3'end. Widespread expression of natural antisense transcripts (NAT) has, in recent times [17-19], emerged as a potential mechanism for bringing diversity and regulatory complexity to a surprisingly finite human 'protein coding' genome [12]. While there are a handful of examples of NAT (coding or non-coding) which negatively regulate protein coding mRNA expression [20], few are associated with human disease [19] and none have been shown to directly regulate a *cis*-transcribed partner in a positive or concordant manner. In addition, despite the annotation of several thousands of ncNAT's in mammalian genomes, few have had their role in mammalian biology clarified [17,20].

Greater appreciation of the mechanisms responsible for regulating the *PINK1 *gene locus, beyond rare mutations, is of importance for a number of reasons. For example, it is likely that alteration of PINK1 function, regardless of mechanism, would influence mitochondrial function [1,10-12,21]. In addition, as PINK1 appears to influence mitochondrial membrane potential [1] and mitochondrial dysfunction is implicated in many age related diseases [22], studying the regulation of the *PINK1 *locus during dynamic *in vivo *modulation of mitochondrial function is likely to be relevant to human ageing. While it is impossible to study this process in the adult human brain, it is plausible to model gains in mitochondrial function in adult human skeletal muscle [23] and examine the regulation of transcription from the *PINK1 *locus. In doing so we reveal direct evidence that there is a discordant transcriptional response between the two coding PINK1 splice variants, *in vivo*, during dynamic alterations in mitochondrial function, whereas there is a concordant regulation of short transcript variant of PINK1 (svPINK1) by a *cis*-transcribed ncNAT (naPINK1) originating from the *PINK1 *gene locus. Not only do we provide the first direct evidence that a ncNAT can positively regulate the abundance of a sense coding transcript in humans under *physiological *conditions, we also highlight that the complexity of *PINK1 *locus regulation is potentially greater in humans than other species.

## Results

### Analysis of the *PINK1 *locus

**Figure 1:**
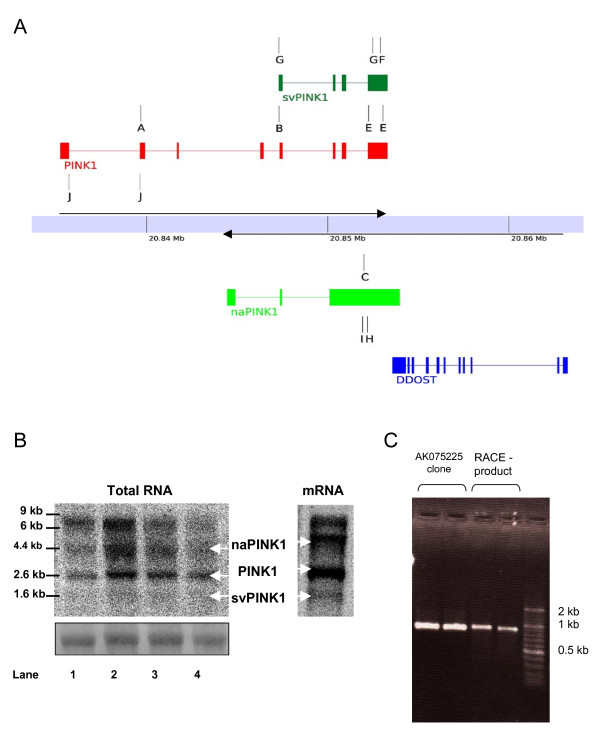
Mapping out the *PINK1 *locus **(a)**. To validate the predicted transcripts from the *PINK1 *locus, Northern blotting and 5'-RLM RACE were performed **(b-c)**. **(a) **A scaled overview of the *PINK1 *locus, presented along with the precise location of primer sites, siRNA target sites and Northern probe locations. The diagram represents the chromosomal coordinates for the PINK1 gene annotated in the Ensembl gene browser (20,832,535–20,850,591, chromosome 1). Arrows indicate direction of transcription (i.e *PINK1 *and *svPINK1 *are transcribed from left to right, while *DDOST *and *naPINK1 *are transcribed from right to left. **(b) **Total RNA was isolated from 4 neuroblastoma cell lines (SH-SY5Y (1), SK-N-SH (2), SK-N-AS (3), SK-N-F1 (4)) and poly adenylated mRNA was isolated using an oligo d(T) magnetic bead kit (from a pool of total RNA from all cell lines) and analyzed by Northern blotting with a double stranded probe targeting PINK1 exon 8, and thus would detect naPINK1 (4.4 kb), PINK1 (2.6 kb) and svPINK1 (1.6 kb). Three bands were detected of which two of them corresponded to the sizes of naPINK1 and PINK1, respectively. The third band is between 6 and 9 kb. It is plausible that it represents a larger transcript consisting of both the DDOST and the naPINK1 transcripts, which would result in a transcript of 6.4 kb. A fourth, band was detected in the mRNA preparation with a size that corresponds to svPINK1 (1.6 kb) consistent with the qRT-PCR abundance of svPINK1 **(c) **5'-RLM RACE was performed to clone svPINK1 full-length cDNA. The svPINK1 RACE-product was confirmed using nested PCR. In parallel, as a size control, the same nested PCR was performed on a purchased clone (AK075225) containing the svPINK1 sequence. This clone was found using BLAST at the NCBI database. The size of the PCR products were determined by agarose gel analysis, which demonstrated bands at ~854 bp for both templates, consistent with the predicted size of the svPINK1 sequence, and the identify of both bands was then verified by sequencing.

Inspection of the *PINK1 *locus (Fig. 1A) revealed a potential for multiple PINK1 mRNA species. In addition to the protein-coding PINK1 transcript, 2.6 kb long, the *PINK1 *locus encodes a shorter transcript, svPINK1, which almost completely overlaps with the 5'end of a *cis*-transcribed natural antisense transcript, naPINK1 and also overlaps with the 3' portion of the full length PINK1 transcript. svPINK1 is 1.6 kb long and has a unique 52 bp within its first exon derived from the neighboring intronic region [24]. This PINK1 splice variant represents the C-terminus portion of the PINK1 protein. A model of the PINK1 protein structure was designed using homology modeling (see Additional file 1) [25] where the region colored in grey represents a predicted protein encoded by svPINK1 ([26], Q9BXM7-2) which retains a phospho-threonine binding domain and a significant portion of the protein kinase domain (see Additional file 1). The 4.4 kb naPINK1 transcript (Fig. 1A and 1B) is annotated as originating from the 'intergenic' region downstream of the 3' end of the protein coding gene *DDOST *(which resides on the opposite strand from *PINK1*). The predicted 5' end of naPINK1 overlaps with the 3'UTR of DDOST and this region contains no obvious promoter for naPINK1. As the naPINK1 transcript appears to be an extension of the DDOST transcript, this raises the possibility that the promoter for naPINK1 either resides within DDOST or it is regulated by *DDOST *promoter. Interestingly, there was no evidence, across seven species, for conservation of naPINK1, even in the well characterized mouse genome [15,27] or within a recently released natural antisense transcript database [28].

An alignment of the 5' upstream regions (promoter) of *PINK1 *in mouse and human indicate a high degree of conservation, implying that *PINK1 *transcriptional regulation may be similar across these species. A search for transcription factor binding sites within the *PINK1 *5' region [29] identified PPARγ and numerous myogenic transcription factor binding sites (see Additional file 2), supporting the robust expression of PINK1 in mitochondria rich striated muscle and the recently demonstrated and devastating impact of loss of PINK1 expression in *Drosophila *flight muscle [21]. We also examined the genomic sequences of *DDOST *and *PINK1 *(see Additional file 2). The intergenic region and the last exons of both *PINK1 *and *DDOST *are noticeably less conserved between human and mouse than the rest of the locus (approximately 60% conservation compared with >80%), which supports the idea that the region from where the naPINK1 originates, is specific to the human genome. Interestingly, *DDOST *encodes the AGE-R1 scavenger receptor, a counter regulatory mechanism for advanced glycation end-product induced oxidative stress [30], with a role in diabetes and ageing, processes clearly relevant to Parkinson's disease [8]. Evidence that this 'intergenic' region between the 3' end of DDOST and the 5' end of naPINK1 is subject to post-transcriptional regulation comes from the observation that between the 3' end of DDOST and the 5' end of naPINK1, there are numerous consensus sequences for endogenous microRNA's (see Additional file 2) providing a potential mechanism through which the naPINK1 transcript could be processed from a large combined transcript (see below).

### Detection of the RNA transcripts from the *PINK1 *locus

We profiled, using Northern blot, a range of neuroblastoma cell lines to look for divergent expression of the *PINK1 *related transcripts (with a view towards future cell studies). Although no major differences between the cell lines were detected, we confirmed expression of PINK1, and produced the first direct evidence for the existence of naPINK1 (Fig. 1B). svPINK1 was clearly of low abundance (consistent with the estimated 20 fold lower abundance using quantitative real-time PCR (qRT-PCR) specific primer/probe based detection (Table 1). This transcript was poorly visible by Northern blot of total RNA, but became clearly detectable when using mRNA as the starting material (Fig. 1B). To confirm the existence of the predicted svPINK1 transcript, we used 5' RLM RACE and then cloned and sequenced the product, along with a purchased cDNA clone (AK075225), representing the svPINK1 genomic location. This strategy enabled rapid verification that we had the correct size of product on an agarose gel (Fig. 1C), before proceeding with sequencing. In addition we used both random hexamer and Oligo d(T)_16 _priming strategies to detect PINK1, svPINK1 and naPINK1 expression in neuroblastoma cell lines using qRT-PCR (see below). Detection of identical abundances of svPINK1 and naPINK1 with both primer strategies indicated that both svPINK1 and naPINK1 are polyadenylated (data not shown). All primers and probes used in this study can be found in Table 1 and there relative location is illustrated in Fig. 1A.

### *In vivo *human transcript regulation of the *PINK1 *genomic locus

**Figure 2:**
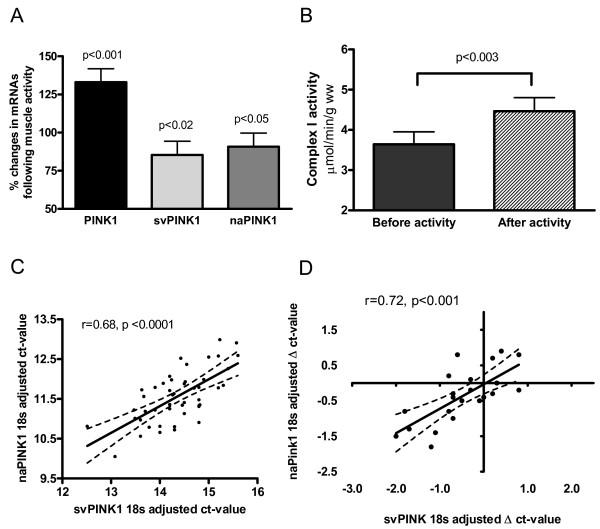
Expression of PINK1, svPINK1 and naPINK1 in humans *in vivo *during gain in mitochondrial activity induced by 6 weeks of endurance training **(a-d)**. Total RNA was isolated from human muscle biopsies before and after activity to manipulate mitochondrial content. Expression of PINK1, svPINK1 and naPINK1 was achieved using quantitative real-time PCR (qRT-PCR). Mitochondrial Complex I activity demonstrates gain of mitochondrial content. Data are mean ± se and are presented as a percentage of the expression (or enzyme activity) before and after activity. **(a) **PINK1, svPINK1 and naPINK1 expression was determined after gain in mitochondrial activity (n = 24 for PINK1 and naPINK1, n = 23 for svPINK1 as the expression of svPINK1 in one paired sample was to low to measure). **(b) **Mitochondrial complex I activity and MTND4 mRNA were measured in muscle biopsies (n = 24) and the increase in enzyme activity is presented **(c) **Linear regression and correlation analysis of naPINK1 expression *versus *svPINK1 expression in human *in vivo*. Values compared were 18S rRNA adjusted CT-values from qRT-PCR analysis (n = 48). Dotted lines represent 95% confidence intervals. **(d) **Linear regression analysis of the changes in naPINK1 expression *versus *the changes in svPINK1 expression in the human *in vivo *model for mitochondrial biogenesis. Values compared were from the qRT-PCR analysis (18S adjusted CT-values before subtracted from 18S adjusted CT-values after 6 weeks of endurance training, n = 24).

If PINK1 plays a physiological role in human mitochondria then its expression must be regulated in a manner that is consistent with dynamic changes in mitochondrial function. Convincingly, we demonstrate dynamic up-regulation of PINK1 expression during gain of mitochondrial function (Fig. 2A–B). There was also a modest but significant correlation (r = 0.34, p = 0.018) between the mitochondrial DNA (mtDNA) encoded complex I gene, MTND4 expression and PINK1 mRNA. *In vivo*, svPINK1 demonstrated a diametrically opposed expression pattern to PINK1 (Fig. 2A). This suggests that splicing is somehow influenced by a requirement for PINK1 within the mitochondria. Strikingly, we observed *in vivo *an extremely robust positive co-expression of svPINK1 mRNA and naPINK1 (Fig. 2C). Thus, the change in svPINK1 expression was *proportional *to the change in naPINK1 expression *in vivo*. Moreover, there was also a strong positive covariance within individual human subjects for the *changes *in svPINK1 and naPINK1 mRNA expression (Fig. 2D) providing an additional and highly convincing indication that naPINK1 may regulate svPINK1 abundance *in vivo *at physiological levels. In stark contrast, PINK1 does not co-vary with naPINK1 *in vivo*, while the ratio of PINK1 to svPINK1 is associated with the NAT expression pattern (see Additional file 3). Hence, only the sense coded mRNA that demonstrates near fully complementary overlap with the NAT appears co-expressed *in vivo*. Thus we speculate that *PINK1 *may play a physiological role in regulating mitochondrial oxidative phosphorylation (consistent with articles published during the review of our manuscript, [10-12]), while svPINK1, which is homologous to the C-terminus end of the full-length protein, may regulate PINK1 kinase function [2].

### RNAi demonstrates that naPINK1 regulates svPINK1 abundance

**Figure 3:**
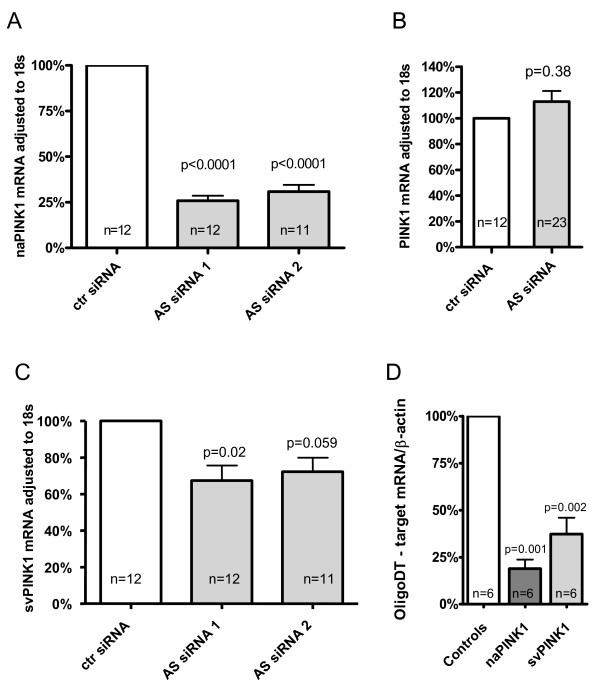
Expression of naPINK1, PINK1 and svPINK1 following knockdown of naPINK1 with short interfering RNA (siRNA) **(a-d)**. Total RNA was isolated from neuroblastoma cell lines SK-N-MC and SH-SY5Y treated with two different siRNAs towards naPINK1 or an siRNA control which avoids targeting any known human sequence. Random hexamers were used in cDNA-synthesis when not otherwise stated and gene expression was determined using qRT-PCR. Data are presented as mean ± SE of the percentage mRNA abundance related to control siRNA treated samples in each experiment. **(a) **naPINK1 expression following AS-siRNA knockdown (n = 12 for control siRNA and AS siRNA 1, n = 11 for AS siRNA 2, one sample was excluded due to failed transfection). **(b) **PINK1 expression following AS-siRNA knockdown (n = 12 for control siRNA and n = 23 for pooled results from the two siRNAs). **(c) **svPINK1 following AS-siRNA knockdown (n = 12 for control siRNA and AS siRNA 1, n = 11 for AS siRNA 2). **(d) **To assess if naPINK1 and svPINK1 were polyadenylated and to exclude amplification of pre-mRNA species, naPINK1 and svPINK1 expression was determined in a subset of samples (n = 6) from which an additional cDNA synthesis was performed, this time using oligo d(T)_16_. Absolute values obtained for gene expression were similar to the random hexamer protocol while the extent of the interaction between naPINK1 and svPINK1 was arguably clearer.

To explore the natural antisense relationship further, we used short interfering RNA (siRNA) directed specifically towards naPINK1 or PINK1 (Table 1 and Fig. 1A). Targeting of the naPINK1 transcript in neuroblastoma cells had no significant effect on the abundance of the PINK1 transcript (Fig. 3A–B). In stark contrast, svPINK1 was reduced by 40% following selective knockdown of the *cis*-encoded antisense transcript naPINK1 (Fig. 3C). Note that we used siRNAs that targeted unique, non-overlapping, regions of the 4.4 kb sequence of naPINK1 (siRNAs H and I, Table 1). These siRNA target an intronic sequence which can be found in the unprocessed svPINK1 and PINK1 sequence but which is within the mature naPINK1 mRNA sequence. Although RNAi is supposed to follow an asymmetry rule [31] it is possible that the guide siRNA could also direct RNAi – against the intronic unprocessed PINK1 variant. Not only do we think this is highly unlikely, but our data directly demonstrate that this does not occur in the present situation as such an action would result in knockdown of both svPINK and PINK1, and this clearly does not occur (compare Fig. 3B and Fig. 3D). When cDNA was synthesized using oligo-d(T)_16 _primers, svPINK expression was reduced by ~60% following knockdown of the naPINK transcript with AS siRNAs (Fig. 3D), consistent with data generated using random hexamer priming (Fig. 3C). In addition, siRNA targeting a region on the first exon of PINK1 (S PINK1-1) robustly knocked down PINK1 mRNA but this did not significantly alter either svPINK1 or naPINK1 expression (see Additional file 3). Thus, it appears that when siRNA mediated cytosolic knockdown of the polyadenylated naPINK1 transcript occurs, svPINK1's abundance is reduced, which in turn strongly supports the above concordant *in vivo *human interactions between PINK1 mRNA and its ncNAT.

## Discussions and conclusion

In the present study we demonstrate that a naturally expressed non coding antisense RNA and a short splice variant of PINK1 exist in human neuroblastoma cells and human skeletal muscle tissue. We further demonstrate that this NAT regulates the *PINK1 *locus in a manner that involves stabilizing, or promotion of, the expression of the PINK1 splice variant, svPINK1. This is the first compelling evidence that an ncNAT positively modulates a sense coding RNA at levels found *in vivo *in humans. Furthermore, given that svPINK1 codes for a homologue of the C-terminus of PINK1; a peptide sequence which regulates PINK1 kinase activity [2], then modulation of naPINK1 expression may have direct relevance in Parkinson's disease.

Teleologically, any process that regulates *PINK1 *activity may influence Parkinson's disease progression and thus it is important to consider the broader regulation of the *PINK1 *locus. The scale of the FANTOM Consortium mammalian antisense transcriptome project [17] enabled robust estimations, using gene ontology based classification scores, of the function of the coding genes which have *cis*-transcribed associated NATs. For convergent NAT species, the corresponding coding transcripts were most likely translated into catalytic proteins involved in nucleotide metabolism, while for fully overlapping *cis*-transcribed NAT species, many of the corresponding sense mRNAs were translated into a protein with protein binding abilities or kinase activity. These statistical associations are clearly in agreement with the naPINK1 and PINK1/svPINK1 relationships. In the present study we provide robust evidence of regulation by the naPINK1 transcript of the short variant of the Parkinson's disease gene, *PINK1*, under conditions where the mRNA species do not exceed their natural abundances (i.e. we do not rely on artificial over expression to study these RNA interactions).

A further point of interest is that the naPINK1 transcript is formed at a region concurrent with the 3' end of the *DDOST *gene. Examination reveals no obvious promoter region within the overlapping region that encodes for naPINK1 and the 3'UTR of *DDOST*, and so we can speculate that naPINK1 and *DDOST *are transcribed from a common promoter and that naPINK1 is a result of transcription read-through. Our Northern blot analysis (Fig. 1B) supports this idea as we found a large transcript (6–7 kb) in both the total RNA and isolated mRNA preparations that could represent a DDOST-naPINK1 transcript (which should be 6.4 kb). It has been postulated that neurodegenerative diseases, such as Parkinson's, are associated with oxidative stress [7,8] and there is further association between metabolic syndrome and neurodegeneration [32-34]. *DDOST *encodes a receptor that mediates suppression of advanced glycation mediated cellular oxidative stress and apoptosis [30]. While transcriptional modulation of the *DDOST *gene has not been studied, it's possible that metabolic syndrome induced transcriptional modulation of *DDOST *could subsequently result in altered naPINK1 expression.

We must also ask how naPINK1 is able to 'stabilize' the svPINK1 transcript. In general, the precise molecular mechanisms through which ncNAT leads to the regulation of a sense mRNA have not been clarified [17,19,12,35]. RNA duplex formation leading to RNA editing or degradation has been suggested, while antisense induced chromosomal modifications has been demonstrated and result in gene silencing. For example, the murine ncNAT *Air *[36], which regulates the maternally expressed igf2 receptor gene (*igfr*), highlights a multi-gene interaction and provides a clear indication of the complexity of NAT regulated transcription. Although only over-lapping *igf2r*, the paternal antisense transcript *Air*, silences the expression of *igf2r*, *Slc22a2 *and *Slc22a3 *through an ill-defined epigenetic modification [37]. There are also examples where the antisense transcript regulates transcription (*N-myc*), inhibits splicing (*Rev-erbAα*), transport (*mld*) or promotes degradation (*bFGF*). These examples all involve a *reduction *in mRNA expression [35]. A short co-transcribed ncNAT from the *WT1 *gene loci has been associated with *greater *abundance of *WT1 *protein [38] yet *in vitro *mechanistic studies failed to clarify this effect. In our case, naPINK1 positively regulates the abundance of svPINK1, both *in vivo *and during siRNA knockdown experiments and thus we present a novel observation for a ncNAT. Importantly, we demonstrate that the detectable levels of naPINK1 and svPINK1 are likely to be mature polyadenylated transcripts and so the strong *in vivo *association may reflect a cytosolic interaction. Insight into the mechanism through which naPINK1 may regulate svPINK1 also comes from the observation that naPINK1 appears only to influence the abundance of the near full length complementary *cis *transcribed svPINK1 sequence (only 52 bases of the 5' end of svPINK1 are non-complementary) suggesting a potential role for dsRNA mediated stabilization. Recent data from chloroplasts, involving the mitochondrial ATP synthase gene (*atpB*), demonstrated that a chloroplast mutant that produced a complementary coded *atpb *NAT mRNA resulted in dsRNA formation, which *prevented *exonuclease mediated degradation of the *atpB *mRNA [39]. It is also thought that the degree of RNA editing, modulates activity of the RNAi pathway, and it is plausible that this might explain the current observations.

It was recently reported that siRNA mediated transcript degradation can act in the nucleus of mammals [40], however this was demonstrated using a high concentration of a siRNA containing a sequence known to result in off-target effects [41]. In our experiments, RNAi directed degradation of naPINK1 is most likely to occur in the cytosol, and we clearly demonstrate that this causes a significant reduction of svPINK1. One could argue that naPINK1 may influence the splicing of PINK1 pre-mRNA. Under this scenario, association between the 5' end of the PINK1 pre-processed mRNA with the natural antisense molecule would somehow favor the alternative splicing process to produce svPINK, starting from the intronic region between Exon 4 and Exon 5 of PINK1, implying that naPINK1 potentially masks a splicing inhibitor site. In agreement with this idea, if one examines the *in vivo *relationship between the ratio of PINK1:svPINK1 RNA then this is also linearly related to the expression level of the naPINK1 (see Additional file 3). Nevertheless, this correlation is driven by the svPINK1-naPINK1 relationship and it is clear that PINK1 itself does not co-vary with the naPINK1 RNA under the present experimental conditions (see Additional file 3). Given that we demonstrate that siRNA H and I do not target the pre-spliced version of svPINK1 or PINK1 (which prior to nuclear processing and export contain a common intronic region identical to the siRNA targeted region on the naPINK1 transcript) and that is possible that svPINK1-naPINK1 relationship reflects alternative splicing, then we have to consider that the synthetic siRNA may indeed direct RNAi within the nucleus (naPINK1 knock-down) with a subsequent change in PINK1 splicing, a consideration that merits more robust investigation.

The regulation of PINK1 protein expression is likely to be complex, particularly under disease conditions [9-12,42]. Available evidence suggests that the full length protein is processed (removal of its mitochondrial localization tag [2,9]), resulting in the detection of at least two proteins on a western blot. Neither of these proteins is consistent with the predicted size of a svPINK1 protein (they are both too large). Multiple shorter proteins have been found when GST-tagged PINK1 is over-expressed and detected using an antibody to GST [43] and while others have presented western blots demonstrating a single protein band [44], so far all commercial antibodies that we purchased reproducibly (or received as a gift) give rise to >8 discrete bands and thus are not reliable when profiling cell models. As more reliable reagents become available [9] it will become important to combine well designed RNAi experiments with protein detection, to study the biochemical consequence of loss of PINK1, svPINK1 and naPINK1 in human cells. From a speciation perspective, there is prior evidence that natural antisense RNA sequences can be conserved selectively in higher mammals. A *cis*-transcribed intronic antisense mRNA from the FAS receptor gene, named *Saf*, alters FAS splice variant expression when *Saf *is artificially over-expressed [45] and *Saf *appears only to be conserved in primates. Future experiments will also have to address the relative importance of naPINK and svPINK in relation to PINK1 function. For example, the relative abundance of naPINK approaches that of PINK1 in tumor cell lines, while naPINK1 is less abundant, although clearly present in human muscle tissue. Ongoing svPINK1 over-expression studies should resolve the function of svPINK1, while we must also decide whether svPINK1 is a truly independent 'gene' transcribed from within the PINK1 genomic loci. In addition, given the complexity of defining what merits being called a 'gene' it is reasonable to suggest that a NAT, with a proven biological function, should also be considered a gene.

In summary, we produce *in vivo *human data and supportive RNAi data which demonstrates that a non-coding RNA so far limited to human expression, appears to 'stabilize' a sense coding RNA. While our data is the first evidence that endogenous levels of a non-coding antisense RNA influencing a sense coded mRNA in a concordant fashion, we can not rule out the possibility that naPINK1 recruits additional cytosolic proteins [20] to bind and stabilize svPINK1 in a more complex scenario than direct complementary hybridization. Given the close genomic association between DDOST and naPINK specifically in the human genome, we speculate that these two disease related genes may be co-regulated, and uniquely so in humans.

## Methods

### Cell culture and transfection

All cell culture reagents were purchased from GIBCO Invitrogen (Sweden) unless otherwise stated. Neuroblastoma cell lines SK-N-MC, SK-N-DZ, SK-N-F1, SK-N-AS were sub-cultured in Minimal Essential Media (MEM) + Earle's supplemented with 10% Fetal Bovine serum (FBS), 2 mM L-glutamine, 1 mM sodium pyruvate, 1 × non-essential amino acids, 100 U/ml penicillin and 100 μg/ml streptomycin. Neuroblastoma cell lines SH-SY5Y and SK-N-Be_(2) _was sub-cultured in 50% MEM + Earle's, 50% F12 (HAM) supplemented with 10% FBS, 2 mM L-glutamine, 1 mM sodium pyruvate, 1 × non-essential amino acids, 100 U/ml penicillin and 100 μg/ml streptomycin. Neuroblastoma cell line SK-N-SH was sub-cultured in RPMI 1640 supplemented with 10% FBS, 2 mM L-glutamine, 1 × non-essential amino acids 100 U/ml penicillin and 100 μg/ml streptomycin. Cell cultures were maintained at 37°C in 5% CO_2_.

Prior to transfection, cells were counted using a hemacytometer and 50,000 SK-N-MC cells or 70,000 SH-SY5Y cells were seeded per well, into 6-well plates, 48 hours or 24 hours before transfection, respectively. SK-N-MC attach more slowly to the surface after splitting than SH-SY5Y and are sensitive to transfection if confluence is less than 75%. On the other hand, transfection efficiency of SH-SY5Y was improved by reducing the seeding density. Transfection was performed using 0.25% Lipofectamine 2000 (Invitrogen, Sweden) and 20 nM siRNA in 2.5 ml serum-containing cell culture media (without antibiotics) according to the manufacturer's protocol. Briefly, Lipofectamine 2000 was pre-mixed with Optimem, incubated for 5 minutes and then mixed with siRNA. Following 20 minutes of incubation, the Lipofectamine 2000 – siRNA mix was applied to cells. Media was changed 4 hours later and cells were harvested 48 hrs after transfection for RNA isolation. The siRNAs (Table S1) targeting naPINK1 were designed using an on-line design tool called BLOCK-iT™ RNAi Designer (Invitrogen) and chemically synthesized by Invitrogen. The siRNA targeting PINK1 (Table S1) was a pre-validated siRNA from Ambion (siRNA ID # 1199) and thus designed and chemically synthesized by Ambion. The control siRNA was pre-designed by Ambion to not target any gene in the genome (Silencer^®^Negative Control#1, Ambion)

### RNA isolation and quantitative real-time PCR (qRT-PCR)

Human muscle tissue biopsies were homogenized in TRIzol (Invitrogen) using a motor-driven homogenizer (Polytron, Kinematica) and total RNA was isolated according to the manufacturer's protocol. In brief, two paired samples (before and after activity) were processed in parallel in 1 ml TRIzol at any given time. The sizes of the tissues ranged between 10 and 40 mg. Total RNA was dissolved in 30 μl RNase-free water and quantified using a Nanodrop (NanoDrop Technologies). Typically, the yield was 8–10 μg of total RNA from 20 mg of tissue. For the cell samples, total RNA was isolated and on-column DNase-treated using RNeasy Mini Kit (Qiagen) and RNase-Free DNase set (Qiagen) according to the manufacturer's protocol. Total RNA was eluted in 30 μl of RNase-free water and concentration was measured using a spectrophotometer (UltroSpec 3000, Pharmacia Biotech). The typical yield of SH-SY5Y was 700 ng and of SK-N-MC 5 μg of total RNA per well in a 6-well plate.

1 μg muscle total RNA or 200 ng neuroblastoma cell total RNA, in reaction volumes of 40 μl was reverse transcribed using RT-PCR kit (Applied Biosystems) according to the manufacturer's protocol. Typically, random hexamers were used for the first strand cDNA synthesis. The advantage of using random hexamers is the possibility to use 18S rRNA as an endogenous control in the quantitative real-time PCR (qRT-PCR) analysis. However, to ensure that mRNA and not pre-mRNA was reverse transcribed, a second cDNA synthesis was performed for a sub-set of samples, using oligo d(T)_16 _primer. This was used in an attempt to exclude RNA species lacking poly A tails from being reverse transcribed and only measure processed mature polyadenylated mRNA transcripts in the subsequent qRT-PCR reaction.

Detection of mRNA was performed using an ABI-PRISM^® ^7000 Sequence Detection system (Applied Biosystems, Foster City, CA, USA). Primers and MGB probes (Table 1) were designed using Primer Express software (Applied Biosystems). Primers were synthesized by Invitrogen and MGB probes by Applied Biosystems. A pre-optimized primer and probe assay for 18S rRNA was used as an endogenous control (Applied Biosystems). For cDNA synthesized with oligo d(T)_16_, β-actin was used as endogenous control. Primers and probes were pre-mixed with TaqMan Universal Master Mix (for probes) or SYBR^®^GREEN PCR Master Mix (for primers only) (Applied Biosystems) and applied to 96-well MicroAmp Optical plates (Applied Biosystems). cDNA aliquots of 4 μl were added in triplicates. Muscle cDNA was diluted 1:50 for detection of *PINK1*, 1:10 for detection of svPINK1 and naPINK1 and 1:500 for detection of 18S rRNA. Cell cDNA was diluted 1:2 for detection of all target genes and β-actin and 1:200 for detection of 18S rRNA. Samples were accessed for DNA contamination by including cDNA controls prepared without reverse transcriptase. The amplification of genomic DNA typically amounted to a maximum of <1% of the target gene when using the TRIzol protocol and even less when using RNeasy columns including DNase-treatment. Thermal cycling conditions were: 2 min at 50°C, 10 min at 95°C and 40 cycles of 15 s at 95°C and 1 min at 65°C.

Two-fold dilutions series were performed for all target genes and endogenous controls to determine the amplification efficiency (see Additional file 4). The ΔΔCt method was used to calculate relative changes in mRNA abundance. The threshold cycle (CT) for 18S was subtracted from the CT for the target gene to adjust for variations in the cDNA synthesis. For the *in vivo *samples, the pre-intervention values reflect baseline gene expression levels and were subtracted from the post mitochondrial biogenesis sample (induced under clinical trial conditions using an endurance training model [23] to calculate the increase or decrease in mRNA abundance. For the siRNA experiments, 18S rRNA or β-actin adjusted CT-values were related to the adjusted CT-value of the control-siRNA treated sample in each experiment, transformed from logarithmic to linear and then a mean from all experiments was calculated. The results are presented as percentage mRNA abundance of control siRNA treated samples (mean ± SE).

### Northern blot

Neuroblastoma cell lines SK-N-MC, SH-SY5Y, SK-N-DZ, SK-N-F1, SK-N-AS, SK-N-Be_(2) _and SK-N-SH were harvested from 10 cm culture dishes with 2 ml of TRIzol (Invitrogen) and total RNA was isolated as described in the manufacturer's protocol. A pool of totalRNA from all the above cell lines was used for polyadenylated mRNA isolation using an oligo d(T) magnetic bead kit for mRNA isolation (Promega) Total RNA and mRNA was examined by Northern blotting. 10 μg of total RNA were separated on an agarose gel (1.2%) containing 20 mM MOPS (pH 7.0) and 6.7% formaldehyde. Ethidium bromide was added to the gel to examine the distribution of RNA and to determine the abundance of 18S rRNA abundance across the samples. The gel was run for 5 to 6 hours at 80 V and the RNA was then transferred by capillary blotting overnight onto a Hybond-XL membrane (Amersham Biosciences). The membrane was pre hybridized at 42°C for at least 3 h in hybridization solution containing 5 × SSC (1 × SSC is 0.15 M NaCl/0.015 M sodium citrate), 5 × Denhardt's (1 × Denhardt's is 0.02% Ficoll 400/0.02% polyvinylpyrrolidone/0.02% BSA), 0.5% (w/v) SDS, 50 mM sodium phosphate (pH 6.5), 50% formamide and 100 μg/ml sheared herring sperm DNA. Hybridization of the membrane was performed overnight at 42°C in a hybridization solution with the addition of denatured probe, [^32^P]dCTP-labelled cDNA, corresponding to PINK1 amplified by PCR (primers in table 1) and labeled using a random priming kit (Amersham Biosciences). After hybridization, the solution was removed and the membrane was washed twice for 30 min at room temperature in a solution of 2 × SSC and 0.2% (w/v) SDS and twice for 15 min at 50°C in a solution of 0.1 × SSC and 0.2% (w/v) SDS. The membrane was sealed in a plastic envelope and then exposed and scanned in a Fuji Film Phosphoimager. Specific mRNA signals were analyzed with the Image Gauge V3.45 program (Fuji Film).

### 5'-RACE and cloning of svPINK1

RNA Ligase Mediated Rapid amplification of cDNA ends (RLM-RACE kit, Ambion) was performed according to the manufacturer's protocol in order to clone svPINK1 full-length cDNA. Briefly, total RNA from SK-N-MC cells (isolated with TRIzol in the same way as for Northern blot according to above) was used as template for the reaction. A 45 base RNA adaptor oligonucleotide was ligated to the 5' end of the de-capped RNA population. The RNA was reverse transcribed using RT-PCR kit from Applied Biosystems instead of the reverse transcription reagents included in the kit, as the latter gave a very weak signal. Reverse transcription PCR was performed in a PTC-225 Peltier Thermal Cycler (MJ Research) at 25°C for 10 minutes, 48°C for 30 minutes and 95°C for 5 minutes. PCR was performed with the inner primer towards the ligated adaptor sequence (provided in RLM-RACE kit, Ambion) and the outer gene-specific primer (Table 1) designed downstream of the predicted stop codon for svPINK1. A second PCR analysis was performed using the inner primer towards the ligated adaptor sequence (provided in RLM-RACE kit, Ambion) and the inner gene-specific primer (table 1) designed downstream of the predicted stop codon for svPINK1 (see Additional file 4). The sequence was confirmed by a third PCR analysis using internal primers towards the previously amplified PCR product. The 5' internal primer targets and thus the product include the start of the svPINK1 sequence, containing the 52 bases unique to this splice variant. A clone (AK075225) containing the svPINK1 sequence was identified by database searching, purchased from National Institute of Technology and Evaluation (NITE) Department of Biotechnology Biological Resource Center (NBRC), Kazusa-kamatari, Kisarazu, Chiba, 292-0818, Japan [46] and used as template with the internal primers in parallel with the obtained PCR-product from the RACE experiment. The PCR products were validated by agarose gel analysis which demonstrated bands at ~854 bp for both templates, accordingly to the size of the sequence. The product obtained from 5' RACE as well as the product obtained from the AK075225 clone was cloned using standard techniques and sequence analysis confirmed the identity of these sequences.

### Human in vivo skeletal muscle mitochondrial experiments

The study was approved by the ethics committee of the Karolinska Institute, Stockholm, Sweden, and informed consent was obtained from each of the volunteers. For stimulating increases in mitochondrial function twenty-four subjects trained under full clinical trial conditions on a cycle ergometer as described previously [23]. Physiological determination of increased aerobic capacity and the muscle biopsy procedure were performed as described [23,47]. All muscle biopsy samples were divided into several portions and stored under liquid nitrogen until analysis. RNA analysis was as described above. Mitochondrial enzyme activity was analyzed in whole muscle as described previously. Briefly, muscle biopsies were homogenized with a Potter-Elvehjem glass homogenizer in a KCl buffer (100 mM KCl, 1 mM EDTA, 50 mM Tris, 5 mM MgCl_2_, 1.8 mM ATP, pH 7.2) to obtain a 5% homogenate [48]. For Complex I measurements, 20 μl of whole muscle homogenate was treated with 590 μl disruption solution 1 (5 mM KH_2_PO_4_, 5 mM MgCl_2_, 0.5 g × l^-1 ^essentially fatty acid free bovine serum albumin) and then in 50 μl disruption buffer 2 (7.15 g × l^-1 ^saponin dissolved in disruption buffer 1). Seventy-two μl of the pretreated homogenate was incubated with 75 μl of reagent (50 mM KH_2_PO_4_, 5 mM MgCl_2_, 5 gl^-1 ^BSA, 0.2 mM potassium cyanide, 4.8 μg × ml-1 Antimycine A (Sigma) and 0.12 mM cytochrome c, pH7.5) for 7 min at 35°C. After the incubation, NADH was added to a final concentration of 0.15 mM, and the decrease in absorbance was monitored at 340 nm, for 1 min before and after the addition of rotenone, 2 mg × l^-1^. The rotenone sensitive activity was calculated. The CV for this method is 8.9%.

### Statistical analysis and bioinformatics

All data are presented as mean ± se unless otherwise stated. ANOVA was utilized to analyze the human gene expression responses. When a significant F ratio was achieved, post hoc analysis was utilized to make individual comparisons. For cell based responses, unpaired t-tests were utilized. Sample size and significance level is shown for each graph. The genomic region including the *PINK1 *and *DDOST *genes was obtained from Ensembl v38 for both human (Chromosome 1: 20825000 – 20865000 bp) and mouse (Chromosome 4: 137680000 – 137710000 bp). The human sequence was aligned to the reverse complement of the mouse sequence using Kalign [49]. The alignment was analyzed for conservation using Kalignvu. Transcription factor binding sites were predicted using Consite [50] with a stringent cut-off criteria of 15 bits, using all models in the Jaspar [30, 52] database. A model of the PINK1 protein structure was created using the structure of human protein kinase C theta type (KPCT_HUMAN) in the region 377–625 which is homologous to PINK1 in the region 153–501 [25].

## Abbreviations

ncNAT, non-coding natural antisense; NAT, natural antisense transcripts; svPINK1, short transcript variant of *PINK1*; naPINK1, non-coding natural antisense at the *PINK1 *locus; ORF, open reading frame; qRT-PCR, quantitative real-time PCR; RNA ligase mediated rapid amplification of cDNA ends, RLM-RACE; mtDNA, mitochondrial DNA; RNAi, RNA interference; siRNA, short interfering RNA; MEM, minimal essential media; CT, threshold cycle.

## Authors' contributions

CS carried out the functional genomics studies, the gene expression analysis and participated in drafting the manuscript. NP carried out Northern Blots and RACE. MAF contributed to the idea behind the study and performed initial bioinformatics. TL carried out bioinformatics. KF participated in the gene expression analysis. OR participated in the design of the study and contributed to the gene expression analysis. CW participated in the design of the study and provided useful advises for interpretation of data. LG participated in the molecular biology studies, the design of the study and drafting of the manuscript. JAT conceived of the study and participated in its design and coordination and drafted the manuscript. All authors read and approved the final manuscript.

## Supplementary Material

Additional file 1 Homology modeling of the PINK1 protein.A diagrammatic representation of the PINK1 protein structure using the structure of human protein kinase C theta type (KPCT_HUMAN) in the region 377–625 which is homologous to PINK1 in the region 153–501 [26]. The yellow region is absent in isoform 2 (VSP_050754) and the pink area is exchanged in isoform 2 (VSP_050755). The nucleotide binding sites (red) and phosphoserine site (green) are missing in isoform 2. Click here for file

Additional file 2 Bioinformatic analysis of the *PINK1 *locus. Alignment of the 5' upstream regions (promoter) of *PINK1 *in mouse and human. Prediction of transcription factor binding sites within the *PINK1 *5' region. Examination of the 'intergenic' region between the 3' end of DDOST and the 5' end of naPINK1 identified sequences for endogenous microRNA's. Click here for file

Additional file 3 Identification of svPINK1 transcript by 5'-RLM RACE and qRT-PCR amplification efficiencies. **(a) **First PCR: amplicons were obtained performing PCR with 5'-RACE primer (adapter specific) and gene specific primer (PINK1 specific). Second PCR: 5'-RACE primer (adapter specific) and nested gene specific primer (PINK1 specific). Both amplicons have predicted length (from the beginning of transcript to the end of gene specific primer + the length of adapter amplified by 5'-RACE primer). **(b) **Primer efficiencies are provided for qRT PCR (See Table 1). Click here for file

Additional file 4 Correlations between PINK1, svPINK1 and naPINK1 in human skeletal muscle *in vivo *and cell siRNA experiments. Total RNA was isolated from human muscle biopsies retained before and after 6 weeks of endurance training in 24 subjects. Each subject acted as their own control. This model stimulates mitochondrial biogenesis *in vivo*. cDNA was synthesized and the expression of PINK1, svPINK1 and naPINK1 was determined using qRT-PCR. R values and p values are as indicated in the figures; dotted lines represent 95% confidence interval. **(a) **Linear regression and correlation analysis of naPINK1 expression *versus *PINK1 expression in the human *in vivo *model for mitochondrial biogenesis, demonstrates no association between these two transcripts. Values compared were 18S adjusted CT-values from RT-qPCR analysis (n = 48). **(b) **Linear regression and correlation analysis of naPINK1 expression *versus *the **ratio**of PINK1 and svPINK1 expression in the human *in vivo *model for mitochondrial biogenesis, demonstrates association but is most likely driven by the svPINK1 – naPINK1 association. Values compared were 18S adjusted CT-values from RT-qPCR analysis (n = 48). **(c) **Expression of naPINK1, PINK1 and svPINK1 following knockdown of PINK1 with siRNA. Total RNA was isolated from neuroblastoma cell lines SK-N-MC and SH-SY5Y treated with siRNA towards PINK1 (S PINK1) or an siRNA control not targeting any gene (control siRNA). Random hexamers were used in cDNA-synthesis and gene expression was determined using qRT-PCR analysis. Data are presented as the mean ± se of 18s adjusted Ct value for each transcript. Note that as mRNA abundance declines, then a higher ct (cycle threshold) values is recorded. Each group represents n = 15 independent experiments. Click here for file

## Figures and Tables

**Table 1:** 

**Annotation**	**Description**	**Sequence 5'-3'**
A	PINK1 qRT-PCR forward primer	GGACACGAGACGCTTGCA
	PINK1 MGB probe	TTTCGGCTGGAGGAGTA
	PINK1 qRT-PCR reverse primer	TTACCAATGGACTGCCCTATCA
B	svPINK1 qRT-PCR forward primer	GGGAGTCGTCGATGTGTGGTA
	svPINK1 MGB probe	AGGCCCTCTCCCCTC
	svPINK1 qRT-PCR reverse primer	GGCGCAGGGTACAGGGATA
C	naPINK1 qRT-PCR forward primer	AGCATGCATTGTTGAAATAGCCTTT
	naPINK1 qRT-PCR reverse primer	AGAGGAGACTACTTACCTGGTTCAAG
E	Northern forward primer	CCGCAAATGTGCTTCATCTA
	Northern reverse primer	GGCTAGTCAGGAGGGAAACC
F	5' RACE outer primer	TCCCACTCCCGTAACTGAAC
	5' RACE inner primer	TCCTCAGTCCAGCCTCATCT
G	RACE product internal forward primer	ATTGGGAGTCGTCGATG
	RACE product internal reverse primer	CTCCATGCAGGGACATCA
H	AS siRNA 1 sense strand	GGAAGCUGUAGCUAGAACAUCUGUU
H	AS siRNA 1 antisense strand	AACAGAUCUUCUAGCUACAGCUUCC
I	AS siRNA 2 sense strand	CAGGUAAGUAGUCUCCUCUAUCAUU
I	AS siRNA 2 antisense strand	AAUGAUAGAGGAGACUACUUACCUG
J	S-PINK1 siRNA (spanning two exons) sense strand	GGAGAUCCAGGCAAUUUUUtt
J	S-PINK1 siRNA (spanning two exons) antisense strand	AAAAAUUGCCUGGAUCUCCtg
