# Supplementary figures and images for: The human PINK1 locus is regulated in vivo by a non-coding natural antisense RNA during modulation of mitochondrial function
Source: BMC Genomics. 2007 Mar 15;8:74. doi: 10.1186/1471-2164-8-74 (PMC1831481; doi:10.1186/1471-2164-8-74)

## Slide 1
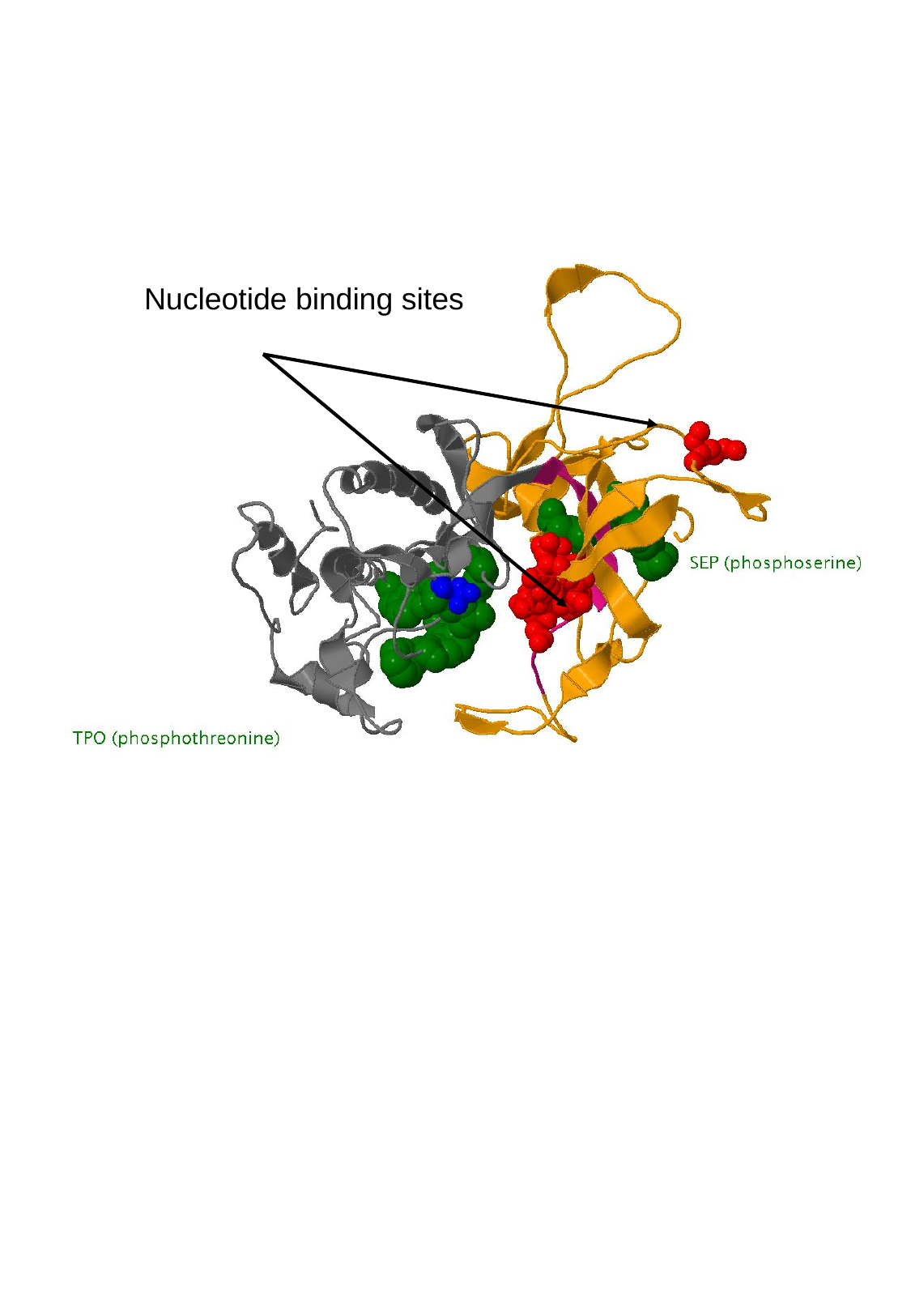

Nucleotide binding sites

Supplement: Additional file 1 — Homology modeling of the PINK1 protein.A diagrammatic representation of the PINK1 protein structure using the structure of human protein kinase C theta type (KPCT_HUMAN) in the region 377–625 which is homologous to PINK1 in the region 153–501 [26]. The yellow region is absent in isoform 2 (VSP_050754) and the pink area is exchanged in isoform 2 (VSP_050755). The nucleotide binding sites (red) and phosphoserine site (green) are missing in isoform 2. [file 1471-2164-8-74-S1.ppt]

## Slide 1
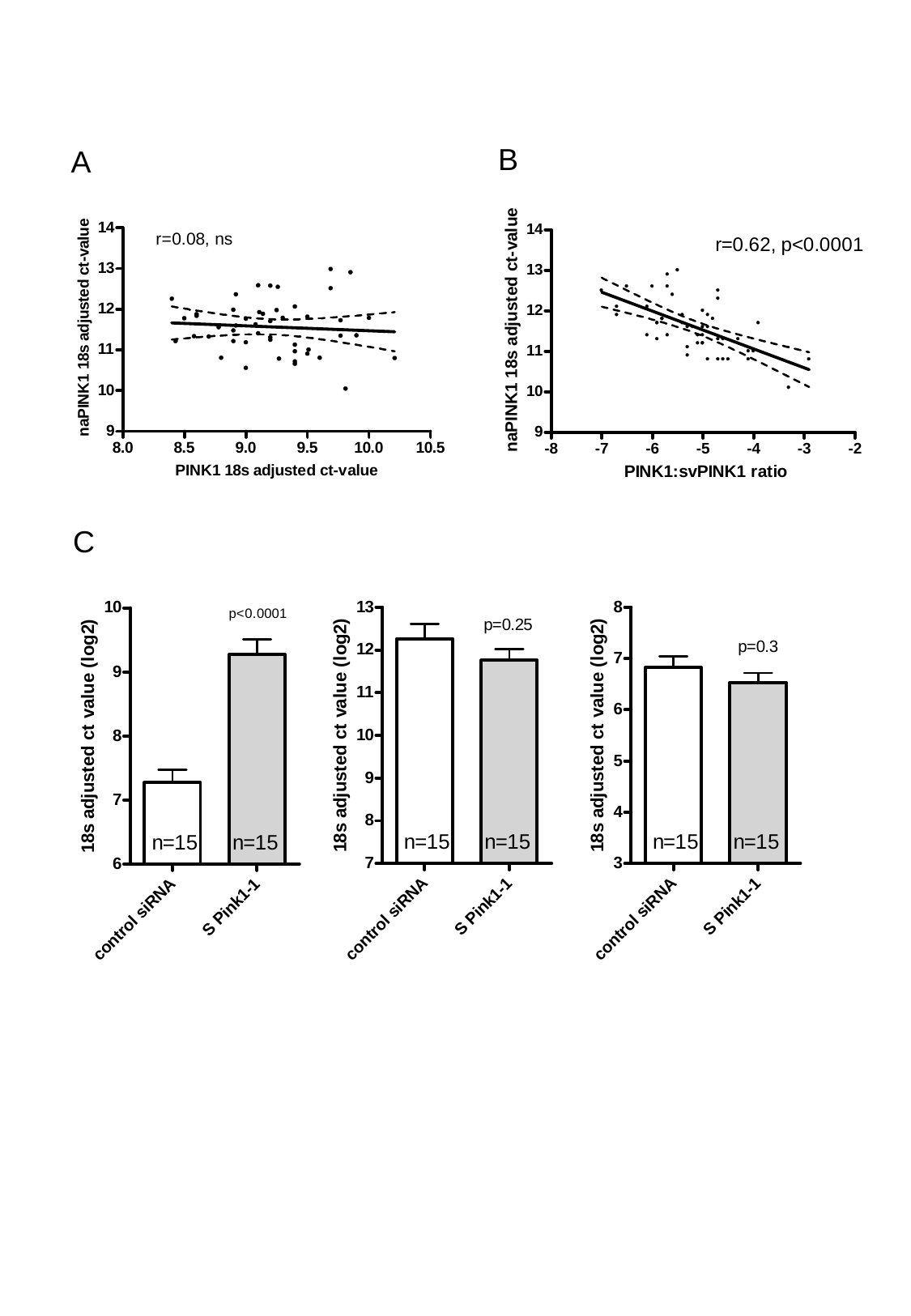

B
A
C

Supplement: Additional file 3 — Identification of svPINK1 transcript by 5'-RLM RACE and qRT-PCR amplification efficiencies. (a) First PCR: amplicons were obtained performing PCR with 5'-RACE primer (adapter specific) and gene specific primer (PINK1 specific). Second PCR: 5'-RACE primer (adapter specific) and nested gene specific primer (PINK1 specific). Both amplicons have predicted length (from the beginning of transcript to the end of gene specific primer + the length of adapter amplified by 5'-RACE primer). (b) Primer efficiencies are provided for qRT PCR (See Table 1). [file 1471-2164-8-74-S3.ppt]

## Slide 1
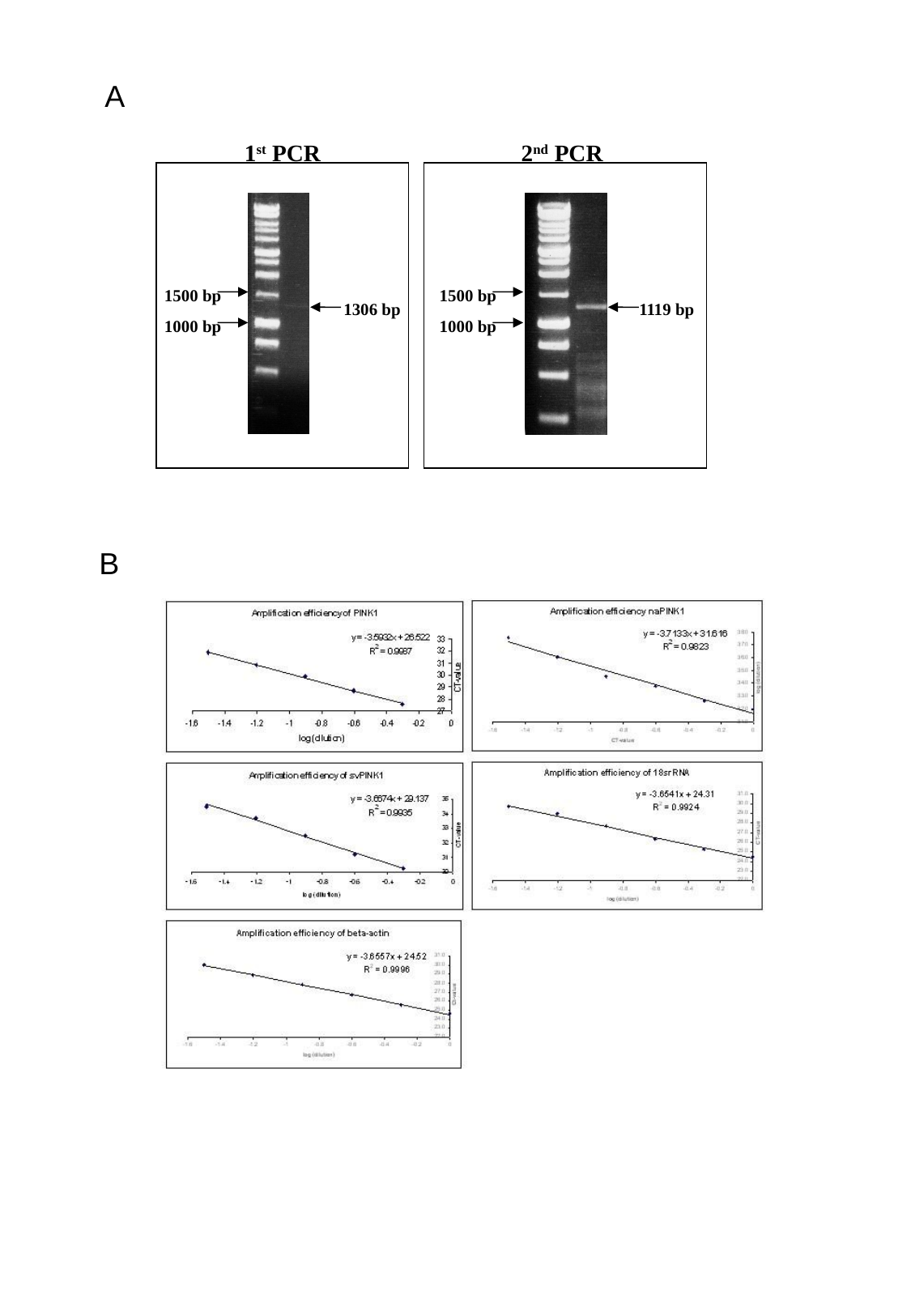

A
1st PCR
2nd PCR
1500 bp
1000 bp
1500 bp
1000 bp
1306 bp
1119 bp
B

Supplement: Additional file 4 — Correlations between PINK1, svPINK1 and naPINK1 in human skeletal muscle in vivo and cell siRNA experiments. Total RNA was isolated from human muscle biopsies retained before and after 6 weeks of endurance training in 24 subjects. Each subject acted as their own control. This model stimulates mitochondrial biogenesis in vivo. cDNA was synthesized and the expression of PINK1, svPINK1 and naPINK1 was determined using qRT-PCR. R values and p values are as indicated in the figures; dotted lines represent 95% confidence interval. (a) Linear regression and correlation analysis of naPINK1 expression versus PINK1 expression in the human in vivo model for mitochondrial biogenesis, demonstrates no association between these two transcripts. Values compared were 18S adjusted CT-values from RT-qPCR analysis (n = 48). (b) Linear regression and correlation analysis of naPINK1 expression versus the ratioof PINK1 and svPINK1 expression in the human in vivo model for mitochondrial biogenesis, demonstrates association but is most likely driven by the svPINK1 – naPINK1 association. Values compared were 18S adjusted CT-values from RT-qPCR analysis (n = 48). (c) Expression of naPINK1, PINK1 and svPINK1 following knockdown of PINK1 with siRNA. Total RNA was isolated from neuroblastoma cell lines SK-N-MC and SH-SY5Y treated with siRNA towards PINK1 (S PINK1) or an siRNA control not targeting any gene (control siRNA). Random hexamers were used in cDNA-synthesis and gene expression was determined using qRT-PCR analysis. Data are presented as the mean ± se of 18s adjusted Ct value for each transcript. Note that as mRNA abundance declines, then a higher ct (cycle threshold) values is recorded. Each group represents n = 15 independent experiments. [file 1471-2164-8-74-S4.ppt]
